# Support for Informal Caregivers in Canada: A Scoping Review from a Hospice and Palliative/End-of-Life Care Lens

**DOI:** 10.1177/08258597221078370

**Published:** 2022-02-24

**Authors:** Andrew Wan, Elaine Lung, Ankita Ankita, Zoey Li, Carol Barrie, Sharon Baxter, Lisa Benedet, Mehrnoush (Noush) Mirhosseini, Raza M. Mirza, Karla Thorpe, Christina Vadeboncoeur, Christopher A. Klinger

**Affiliations:** 1 University of Toronto, Toronto, Ontario, Canada; 2 National Initiative for the Care of the Elderly, Toronto, Ontario, Canada; 3 Canadian Frailty Network, Kingston, Ontario, Canada; 4 Quality End-of-Life Care Coalition of Canada, Ottawa, Ontario, Canada; 5 Canadian Hospice Palliative Care Association, Ottawa, Ontario, Canada; 6 Canadian Home Care Association, Mississauga, Ontario, Canada; 7 College of Family Physicians of Canada, Mississauga, Ontario, Canada; 8 University of Alberta, Calgary, Alberta, Canada; 9 Mental Health Commission of Canada, Ottawa, Ontario, Canada; 10 University of Ottawa, Ottawa, Ontario, Canada; 11 Pallium Canada, Ottawa, Ontario, Canada; 12 McMaster University, Hamilton, Ontario, Canada

**Keywords:** Canada, end of life, (hospice) palliative care, (informal) caregivers, interventions, scoping review

## Abstract

**Objective:** Informal caregivers (ICs) providing care for those at the end-of-life face physical, psycho-social, emotional, and/or financial challenges. However, there is a paucity of research towards the effectiveness of available interventions for this vulnerable population. The purpose of this scoping review was to investigate the availability and efficacy of interventions for ICs providing hospice and palliative/end-of-life care in Canada. **Methods:** Using Arksey and O’Malley's five step framework, a scoping review was conducted in the spring of 2020. Key electronic healthcare, social sciences, and grey literature databases were searched. Relevant publications from 2005 to 2019 were screened for inclusion criteria, and a thematic content analysis was conducted to summarize all key findings. **Results:** Initial searches yielded 145 results out of which 114 distinct articles were obtained. De-duplication and final screening yielded 28 sources which met inclusion criteria (22 peer-reviewed articles [78%] and 6 grey sources [22%]; 12 qualitative papers [42%]). Through thematic content analysis, four major themes were identified: [1] Direct financial support, [2] Direct psycho-sociospiritual support, [3] Indirect patient information provision/education, and [4] Indirect patient support. **Conclusions:** Healthcare practitioners should provide information on patient care and financial aid to ICs. Policies should aim to expand eligibility for and access to financial aid, in particular the Compassionate Care Benefits (CCB). Future research should focus on exploring other interventions, such as physical activities, to better support this vulnerable population. The results from this review will help inform and improve the well-being of ICs providing end-of-life care in Canada and beyond.

## Introduction

People requiring end-of-life (EOL), or hospice and palliative care are often largely supported by informal caregivers (ICs), such as family and friends.^1^ As their health continues to deteriorate, they rely more and more on ICs for assistance in daily activities. However, providing informal care at the end of life comes with its own set of challenges.^2–4^ ICs providing care for EOL patients experience more difficulties compared to non-EOL caregivers.^5^ First off, studies have shown that caregivers providing hospice and palliative/EOL care can suffer from physical challenges, such as physical exhaustion and psycho-socio-spiritual challenges including anxiety and distress.^2–4,6^ Furthermore, ICs often also experience financial strain due to the burdens of caregiving. ICs providing EOL care are more likely to rely on governmental assistance and less likely to receive help from family and friends compared to their non-EOL counterparts.^7^ It has been reported that ICs incur many additional expenses that come along with providing hospice and palliative/EOL care such as transportation, medical equipment, and prescription medication. ICs may also find themselves losing income due to taking unpaid sick days, turning down promotions, or having to quit their jobs altogether.^7^

Unfortunately, due to the emphasis on the patient, it is common for the overall decline in well-being of ICs to pass unnoticed.^3,4^ The importance of ICs in EOL care, as well as the negative health challenges associated with caregiving — including physical exhaustion and mental fatigue —, have become more recognized in the literature, though.^2,3^ Thus, there has been a lot of movement focused on developing interventions (such as community volunteers or the Compassionate Care Benefits (CCB))^6^ that look to alleviate caregiver burden and improve their overall well-being.^8^ However, it remains unclear if current or newly developed caregiving interventions are sufficient and effective for this population. Therefore, it would be valuable to understand how to best support ICs providing hospice and palliative/EOL care by evaluating the current state of support for ICs as their needs can often be missed.^3,4,6^ The purpose of this scoping review was to explore and to examine the availability and effectiveness of current interventions for ICs providing hospice and palliative/EOL care in Canada. Results from this review highlight key concepts alongside knowledge gaps of the current literature and can ultimately help to inform policy, practice, and research on EOL caregiving and improve the overall well-being of ICs.

## Methodological Framework

A scoping review of the literature was conducted following Arksey and O’Malley's five step framework (1. Identifying the research question, 2. Identifying relevant studies, 3. Study selection, 4. Charting the data, and 5. Collating, summarizing, and reporting the results) between December 2019 and April 2020. The goal of a scoping review was to gain a broad understanding of all the available literature related to a topic.^9^

### Identifying the Research Question

The following research question helped to guide this review: *“What are the effects of caregiver support interventions on the physical, psycho-social, and economic health and well-being of informal caregivers providing hospice and palliative/end-of-life care in Canada?”*

### Searching for Relevant Studies

Key electronic healthcare and social sciences databases along with grey literature sources were searched systematically. Advanced Google Search, electronic databases (Ageline, Cumulated Index to Nursing and Allied Health Literature [CINAHL], Cochrane Library, EMBASE, JSTOR, MEDLINE, Project Muse, PsycINFO, Scopus, and Web of Science), and reference lists through the University of Toronto libary system were utilized to search for relevant articles. A combination of the following key terms with Boolean operators (eg, AND, OR, NOT) were used: “informal caregiving”, “(hospice) palliative care”, “caregiver support”, “caregiver interventions”, “physical health”, “psychological health”, “psychosocial health”, “economic health”, and “Canada” (see Supplemental Material). The respective search strings were developed with the help of an experienced librarian.

### Study Selection

Relevant articles were searched and initially screened by five independent reviewers [AA, AW, CAK, EL, ZL]. Appropriate articles for this study were selected using the following eligibility criteria: (1) in English, (2) ICs defined as those providing informal care for hospice, palliative/terminally ill/EOL patients, (3) stems from a Canadian context, and (4) article was published from 2005 onward. Qualitative, quantitative, and grey literature sources were included in this study. Study design/type of article was not part of the inclusion criteria since the goal of this review — in line with Arksey and O’Malley's Framework — was to capture the full breadth of research available for this topic.^9^ The cut-off year for the search (2005) was determined after a straw poll of major databases. The reviewers met to discuss any differences of opinion throughout the screening process and came to an agreement about the final articles included using discussion.

### Charting the Data

Two independent reviewers [AW, EL] scanned the title, abstract, and full-text of all included articles for final study selection. All disagreements were sent to the team and discussed until a final consensus was reached. Selected articles were reviewed and analyzed for data extraction by article information, study design, intervention, outcome measures, findings, and implications for practice, policy, and research (see Supplemental Material), then. Since the aim was to compile all existing literature related to the topic, no appraisal of the quality of the evidence was conducted.^9^

### Collating, Summarizing, and Reporting the Results

A thematic content analysis was performed in line with the research question to describe the results of the review, identifying all descriptions that were related to the topics of “informal caregivers”, “hospice/palliative care”, “interventions”, and “effects”.^10^ Next, similar descriptions were placed into the same pile, with each pile representing initial categories or themes. Categories were then further subdivided to create subthemes. For example, descriptors such as “spiritual”, “journaling”, and “emotional therapy” were categorized under the theme of Direct, Psycho-Socio-Spiritual Support, which was then further subdivided into the subthemes of “Religion”, “Psychoeducation Program”, and “Emotional Support”, respectively. The resulting themes/subthemes were cross-referenced and reoccurring ones containing a high volume of quality material were included (see Supplemental Material). All emerging major patterns, themes/subthemes, and findings were discussed in detail among the team to ensure comprehensiveness and alignment with the overall goals of the scoping review and research question.

## Results

### Search Results

**Figure 1.**
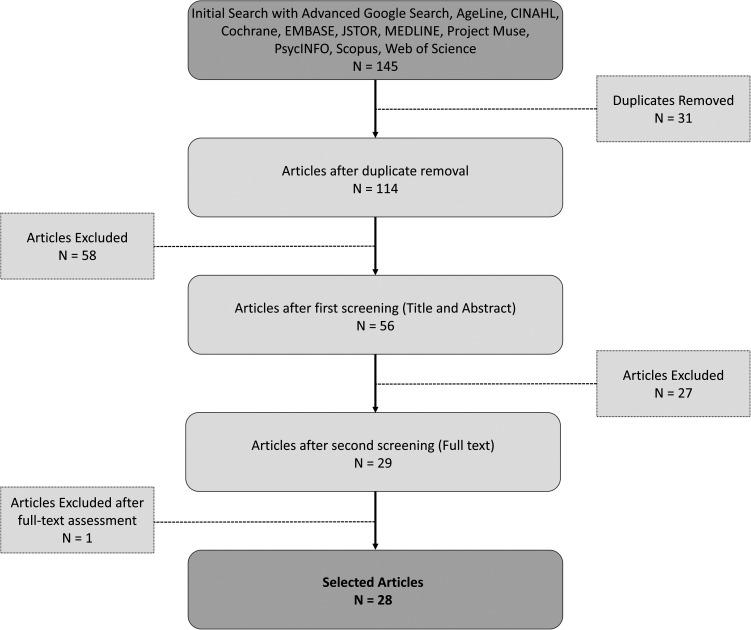
PRISMA flow chart.

**Figure 2.**
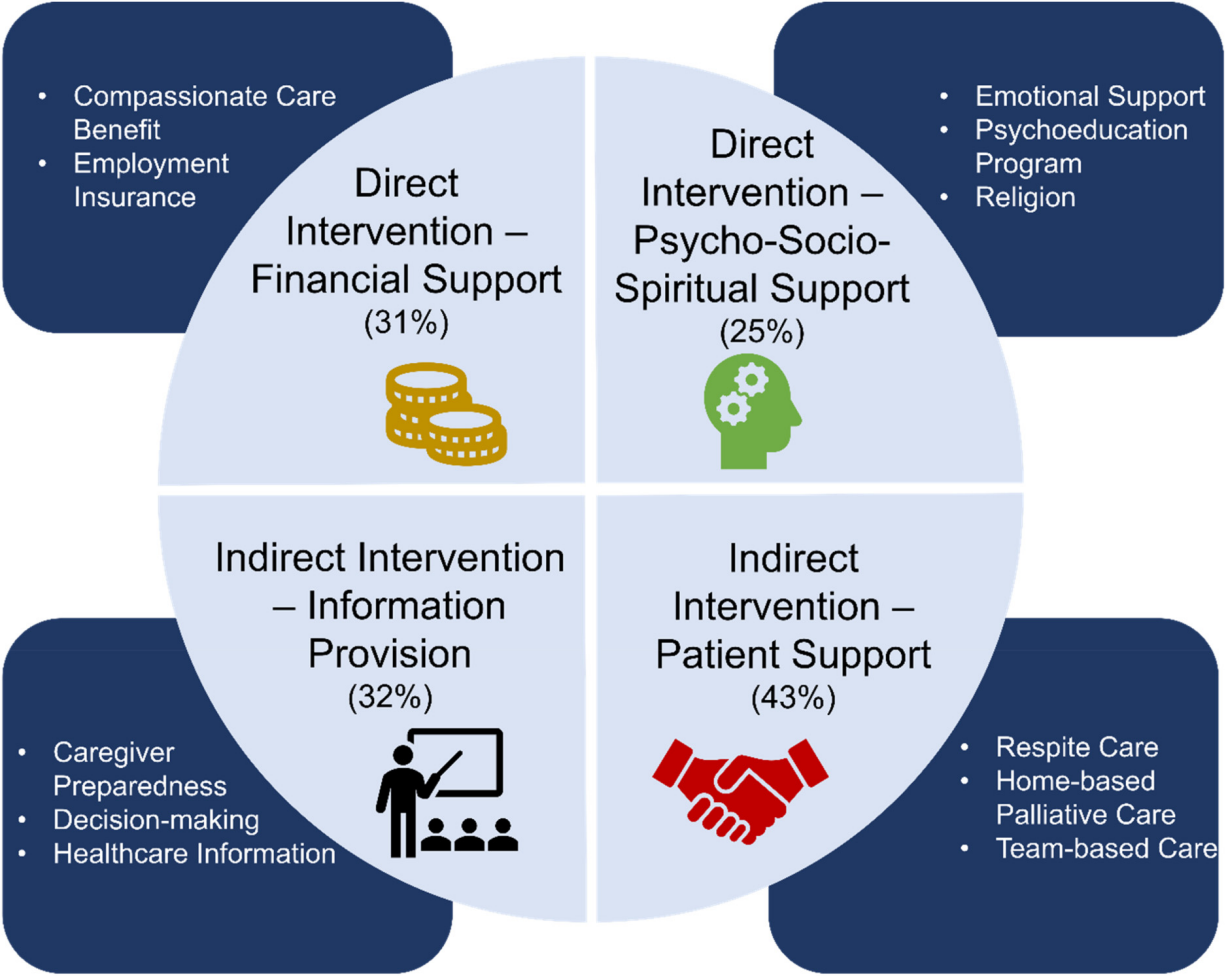
Themes and subthemes emerging from scoping review.

As indicated in Figure 1, initial screening of electronic databases and advanced Google searches yielded 145 articles. After de-duplication and full-text screening, 28 articles were included in this review^11–38^ (22 peer-reviewed [78%]^11–13,15–19,21–25,28–31,34–39^ and 6 grey literature sources [22%].^14,20,26,27,32,33^^)^ The 22 peer-reviewed articles included 12 (55%)^12,13,16,17,19,21,22,28,30–32,38^ qualitative research papers, 4 literature reviews (18%),^18,23,34,36^ 3 mixed-methods papers (13%),^11,25,35^ 1 descriptive study (4%),^24^ 1 longitudinal study (4%),^37^ and 1 randomized control trial (4%).^15^ Four themes were generated as depicted in Figure 2.

### Direct, Psycho-Socio-Spiritual Support

Thirty-two percent of the articles (9/28) included in this scoping review reported the provision of direct, psycho-socio-spiritual support interventions to improve informal caregiving outcomes and experiences.^11–19^

Five articles (18%) suggested that current direct, psycho-socio-spiritual interventions (such as couples therapy) were feasible, adequate, and effective at improving the outcomes for ICs.^11–13,15,17^ One study in particular suggested that emotionally focused therapy, a couples-based intervention, conducted between ICs and their care recipient, provided significant benefits to couples compared with standard care alone.^15^ Results from this study demonstrated that therapy ultimately improved couples’ marital functioning, relational growth, and distress during the end of life.^15^ Benefits related to interventions improving the bond between ICs, their family, friends, and health professionals, were also reported in other studies.^11,13^ Duggleby and colleagues suggested that closer relationships fostered a sense of support and hope that gave ICs the determination and perseverance needed to “survive”.^11,13^ Results from these studies indicated that hope interventions ultimately increased caregivers’ quality of life, overall positivity, and feelings of being valued or heard.^11,13^ One study that evaluated the effectiveness of “death preparedness” interventions also demonstrated improving the relationships and grief responses of ICs providing hospice and palliative care at the end of life.^18^

Other types of interventions were also explored. Journaling was cited as a potentially effective intervention for ICs as it enabled them to have their feelings valued and emphasized the need to better their own health.^11^ A psychoeducation program delivered by social workers for ICs of persons with dementia at the EOL was also found to be effective at reducing stress, enhancing emotional well-being, and strengthening community support.^12^ ICs have also sought out religious and spiritual support. One study noted that many ICs utilized spiritual support and received as much as they desired.^17^ Informal interventions including activities (eg, knitting, sewing, piano) were small escapes and helped ICs to cope with the realities of caregiving.^19^

In contrast, two studies (7%) suggested that current psycho-socio-spiritual interventions did not provide adequate support for ICs.^14,16^ In one study, caregivers wished to have more help processing their emotions and making decisions when faced with uncertainty.^16^ ICs expressed the need for a “coach” who could effectively support and walk caregivers through the whole caregiving process.^16^ *The Way Forward* initiative suggested an integrated approach to care that would enhance caregiver confidence and improve care for palliative care patients.^14^ Some challenges associated with group programs include too much focus on negative aspects of caregiving or participants were simply not ready to open up, which might reduce the effectiveness of such programs. In addition, it could be difficult for ICs to implement these interventions within their already busy schedule.^12^

### Direct, Financial Support

In total, 11 out of the 28 articles (39%) reported on the use of the CCB.^20–30^

The CCB is a federal program designed to financially support ICs providing EOL care to a loved one with a significant risk of death within 26 weeks.^39^ This program is administered as an Employment Insurance (EI) special benefit and applicants must demonstrate that their regular weekly work earnings have diminished by at least 40% due to taking care of their loved ones. The CCB provides financial assistance of up to $573 a week for a maximum of 26 weeks.^39^ Based on the search results, the CCB appears to be one of the few financial assistance programs for ICs providing hospice and palliative care in Canada.

A couple of articles reported on some of the benefits of the CCB for ICs providing EOL care.^27,28^ Among those articles, participants expressed appreciation of the financial support and stated that it helped relieve financial stress. One article noted that successful applicants found the process to be straightforward when they had someone to help them navigate the application. They also felt that they were well supported by coworkers and employers throughout the application process.^27^

On the other hand, nine articles (32%) reported unintended negative consequences with the CCB.^20,21,23–29^ Six articles (21%) discussed the difficulties ICs faced during the application process as they found it to be too lengthy and complicated, especially if English was not their primary language.^20,21,23,24,26,29^ In particular, it was apparent that collecting the necessary letters from employers and physicians for the application was a major hurdle which prevented many applicants from receiving the benefits.^23^ Consequently, ICs felt that the additional paperwork added even more stress during an already busy period of their life, while some reported even feeling isolated.^28^ As such, ICs expressed the desire to be informed of the benefits prior to caregiving through resources such as employers, healthcare providers or even advertising.^25,30^

Another criticism of the CCB was that the time it took for the application to be reviewed and approved was too lengthy. In fact, two articles (7%) stated that the two-week unpaid waiting period added additional financial burden for many families.^21,26^ There were some applicants who already had to quit their job and the waiting period contributed to their growing stress and anxiety.^22^ In certain circumstances, applicants found themselves no longer qualified to receive benefits as their loved one had already passed away as the CCB does not carry over to caregivers during the grief and bereavement period.^24,27^ As such, ICs who required time off work during grief and bereavement did not always feel well supported since there was a lack of government-assisted financial support which might force employees to take unpaid leave.^26,27^ Therefore, the transition back to work for ICs presented yet another period of financial difficulty. For these reasons, some applicants found the CCB to be inflexible and unaccommodating.^22,24^

Lastly, two articles (7%) discussed how successful applicants experienced financial hardship due to caregiving for an extended period of time.^28,31^ ICs found that the CCB did not adequately cover all financial needs (living costs and caregiving costs).^31^ This in fact put them in a more difficult financial situation, as some ICs found themselves without any remaining sick or vacation days and they had to resort to taking unpaid sick leave.^28^

### Indirect Patient Support Interventions

In total, 12 out of the 28 articles (43%) included in this scoping review commented on the effects of providing adequate patient support on caregivers.^12,13,15,16,19,20,26,31–35^ Four articles (14%) noted that better patient support could indirectly benefit ICs and improve their caregiving experience.^13,15,19,31^

Two articles (7%) reported that patients experienced inadequate health services and a slow response for certain provisions.^12,19^ The lack of timely support and limited services resulted in higher distress for ICs. Two articles (7%) investigated the effects of indirect interventions by improving patient care experience such as services to navigate the complex health systems, helping in organizing family affairs, and offering a wide array of support programs.^19,35^ Not only were these interventions important in improving the home-based care experience, but they also helped alleviate caregiver burden experienced by ICs, which improved their overall quality of life.

Respite care is the act of temporarily leaving a person looked after in the care of another party and includes services such as out-of-home respite (eg, around the clock care) or in-home respite (eg, visit from a [health]care provider).^20,26^ These services offer caregivers temporary short-term relief from the physical and emotional demands that come along with caregiving and allow them to attend to their own needs.^20^ One source reported benefits of respite care that included increased satisfaction with life, increased ability to cope, and reduced stress at home for ICs.^20^ However, it seemed that the benefits applied more towards caregivers who had goals for their free time and actively participated in planned activities.^20^ In addition, another source noted that services varied significantly across provinces and territories in Canada, including some regions not providing any in-home respite care.^26^

Three articles (11%) noted that the integration of caregivers into healthcare teams by virtue of training, timely information, and coping strategies showed dual benefits.^31,34,36^ Specifically, interdisciplinary team members were able to address multidimensional needs of the patients and caregivers. The targeted care delivered by this team involved identification of patients most likely to benefit from patient-centred interventions and therefore provided timely care to minimize EOL complications.^36^ A good working relationship between healthcare providers and caregivers added a sense of security that improved the quality of care by helping them understand their role in hospice and palliative care delivery. However, one source found that indirect caregiver interventions provided to the person at the end of life did not have an effect on caregivers’ distress.^33^

### Indirect Interventions: Informational Provision/Education

Thirty-two percent of the articles (9/28) that were included in this scoping review noted the provision of information and education to caregivers about patient's EOL care indirectly improved caregiving outcomes and experiences.^12,19,26,31–34,37,38^

Five articles (18%) suggested direct educational interventions greatly empowered caregivers and increased their quality of life (QoL).^12,31,33,37,38^ Specifically, educational interventions for ICs that were focused on symptom management and coping skills significantly improved informal caregiver QoL.^33,34,36^ ICs perceived some of the benefits of these interventions to include enhanced emotional well-being, increased feelings of preparedness for end of life, comfort, and confidence about their decision making.^12,31,34,37,38^ One particular study found that an educational program that was jointly attended by both bereaved and current caregivers contributed to caregiver's wellness by promoting solidarity, reduced stress, and increased coping.^12^

Despite this, four studies (14%) found the current educational interventions in place were not sufficient and did not adequately support the needs of ICs.^19,25,32,34^ Generally, caregivers wished for more information about death and dying so they could better prepare themselves and family members.^16,19,25,32,34^ In one particular study, ICs expressed the need for concrete information about EOL care so they could “demystify death”.^16^ In addition, many caregivers felt that information about available EOL resources were insufficient and wished this knowledge was provided to them earlier so they could be better prepared.^16,25,32^ As such, three studies (11%) suggested the implementation of education and training from formal healthcare systems to help caregivers better care for hospice and palliative/EOL care patients.^16,25,32^ In particular, one study investigated the potential use of health information technologies such as mobile apps to better deliver palliative care information to caregivers and patients, although the benefits of this technology have yet to be elucidated for ICs.^32^

## Discussion

ICs are typically the main source of support for loved ones at the end of life in Canada. Current literature indicated several challenges that Canadian ICs often face when providing hospice and palliative care.^1–4^ By exploring interventions and their effects on ICs delivering EOL care, several themes were developed that can be used to inform practice, policy, and research in Canada and beyond. Results suggest that current interventions in Canada can be improved to better support ICs providing hospice and palliative care.

### Practice Implications

Similarly to the international literature,^40^ our findings suggest that psycho-socio-spiritual interventions (eg, couples therapy) have beneficial effects for ICs such as decreased distress and increased quality of life.^11–15^ However, results from other studies concluded that current direct psycho-socio-spiritual interventions were ineffective for reducing distress of caregivers in Canada.^12–14^ Thus, our findings suggest that interventions such as psychoeducation programs — which can include regular check-ups with healthcare providers, group counselling, and peer support — may be able to better address the mental and emotional needs of ICs.^12,14,16^ These interventions were shown to improve the overall well-being and to reduce depressive symptoms of ICs and warrant consideration for implementation in the standard of care.^12^

Our findings also suggest that both types of indirect interventions were able to improve ICs’ experiences. Specifically, educational interventions that discussed symptom management for patients were found to enhance the quality of life for ICs. In addition, the integration of patients and caregivers in the healthcare team also contributed to a positive experience for caregivers by enabling more specific interventions and targeted care towards patients. The literature suggests that a combination of educational materials and better patient support can indirectly alleviate caregiver stress and improve the quality of life for both ICs and their loved ones.^31,33,37^ Furthermore, the incorporation of a palliative navigator or better palliative care training for patients and their families can prepare ICs during their caregiver journey.^19,35^ At this time, our findings indicate that there are opportunities to improve current educational interventions and patient support which may lead to better care for ICs and their loved ones.

Currently, the CCB are one of the only sources of financial support that directly aid caregivers in Canada.^39^ Some recipients of the benefits have expressed positive experiences with the CCB as they temporarily alleviated some financial burden.^21,27^ On the other hand, our findings suggest that many ICs were often unaware of the benefits, were not equipped enough to go through the tedious application process or were faced with an abrupt end of the benefits at the time of death of their loved one.

### Policy Implications

At a policy level, the needs of ICs providing EOL care are currently not sufficiently recognized in Canada. Firstly, services such as respite care, day programs, and home care, are often only short-term and do not adequately support long-term caregivers. There is a need to recognize and support a wide array of ICs socially and financially in order to better help them obtain more services and negotiate flexible work hours and space. In addition, changes need to be made to the CCB in order to better accommodate ICs providing EOL care.^12,20,21,24,26,28^ Specifically, policymakers need to think about expanding the criteria to make the benefits more accessible for ICs outside of the work force, expand them into grief and bereavement, and provide more funding in order to help ICs transition back to work more efficiently.

### Research Implications

Findings suggest that current direct psycho-socio-spiritual interventions in Canada have demonstrated some potential in helping ICs. However, more substantial research and larger studies need to be conducted to fully validate the efficacy of these interventions for ICs. In addition, it was noted that all forms of therapies in the literature targeted directly towards ICs were within the domain of psycho-socio-spiritual interventions. It would be interesting to see future research that examined the effect of other forms of interventions on ICs such as physical activities or technology-based interventions (eg, web-based education programs or caregiver apps). Furthermore, future research should also focus on how to best implement and disseminate existing interventions such as the CCB to ICs.^12,21,26,28^

While current literature has suggested that integrating ICs into to the healthcare team can be beneficial for both patients and caregivers, it remains unknown how to best do this. More research is required to investigate what the roles and responsibilities of ICs would be in healthcare teams and how to ensure their needs are met.

### Limitations and Strengths

We have noted several limitations for this scoping review. One limitation is the absence of quality assessment of the included literature. However, based on recommendations of Arksey and O’Malley's framework,^9^ we wanted to ensure that a large range of articles and sources were included in order to obtain a broad understanding of the topic. Another limitation is the selection of only English literature even though Canada is a bilingual country (English and French) due to a limited French comprehension within the research team/no ready access to translators. Lastly, only articles published from 2005 onwards were included for review as we sought to include the more recent literature.

Strengths of our study include exploring the topic within the Canadian healthcare landscape to recognize issues central to Canada and utilizing a comprehensive and systematic methodology to ensure that we obtained and addressed the broadest possible viewpoints. The implications also have relevance to North America and beyond.

## Conclusions

The importance of ICs to people who are dying and to Canada's healthcare systems cannot be overstated. ICs face complex challenges during their caregiver experience as also highlighted in a previous scoping review.^41^ The results of both scoping reviews, when combined, will lead the way toward policy reform by identifying the existing gaps and potential solutions alongside the formulation of new strategies to better support ICs providing EOL care. Ultimately, policy and practice should focus on making current financial aid, education, and psychological interventions more accessible to caregivers. Further research is required to develop and implement effective interventions that are adequately supported for all caregivers. As the complexity of people who need medical care in Canada continues to grow, it becomes even more necessary to ensure that ICs are not forgotten.
